# Phage-display immunoprecipitation sequencing reveals distinct antibody repertoire patterns across rural and urban populations

**DOI:** 10.1016/j.isci.2026.117252

**Published:** 2026-08-18

**Authors:** Ross F. Laidlaw, Gabriel Innocenti, Melanie Prinzensteiner, Thomas Vogl, Moustapha Mbow, Maria Yazdanbakhsh, Mikhael D. Manurung

**Affiliations:** 1 Leiden University Center for Infectious Diseases (LUCID), Leiden University Medical Center, Leiden, the Netherlands; 2 Center for Cancer Research, Medical University of Vienna, Vienna, Austria; 3 Department of Immunology, Faculty of Medicine, Pharmacy, and Odontology, Cheikh Anta Diop University of Dakar, Dakar, Senegal

**Keywords:** phage-display immunoprecipitation sequencing, antibody repertoire, immune variation, multi-omics integration, rural-urban differences

## Abstract

Environmental microbial exposures are hypothesized to shape immune profile differences across rural and urban populations, yet evidence linking exposures to immune phenotypes remains limited. We used phage-displayed immunoprecipitation sequencing (PhIP-seq) to profile antibody repertoires against 357,192 epitopes from >700 organisms in 62 age-matched healthy adults from rural Senegal (*n* = 29), urban Senegal (*n* = 20), and urban Netherlands (*n* = 13). We identified two major patterns of variation: geographically restricted signatures distinguishing Senegal from the Netherlands and an ordered axis spanning rural Senegal, urban Senegal, and urban Netherlands. Rural populations were enriched for antibodies against *Shigella* species and human gammaherpesvirus 8, whereas urban populations showed higher responses against rhinovirus B and *Neisseria meningitidis*. Upon integration of PhIP-seq data with high-dimensional immunological profiling, the antibody repertoire trajectory correlated with a previously established rural-urban immune trajectory and ILC2 frequencies, IgG Fc galactosylation, and serum cytokines. These findings show that antibody repertoire profiling captures distinct microbial exposures across geographic regions.

## Highlights

• PhIP-seq maps antibodies to 357,192 epitopes in 62 rural and urban adults
• Antibody repertoires differ both by region and along a rural-urban axis
• Antibody repertoire correlates with ILC2, IgG galactosylation, and serum cytokines

## Graphical abstract

**Figure:**
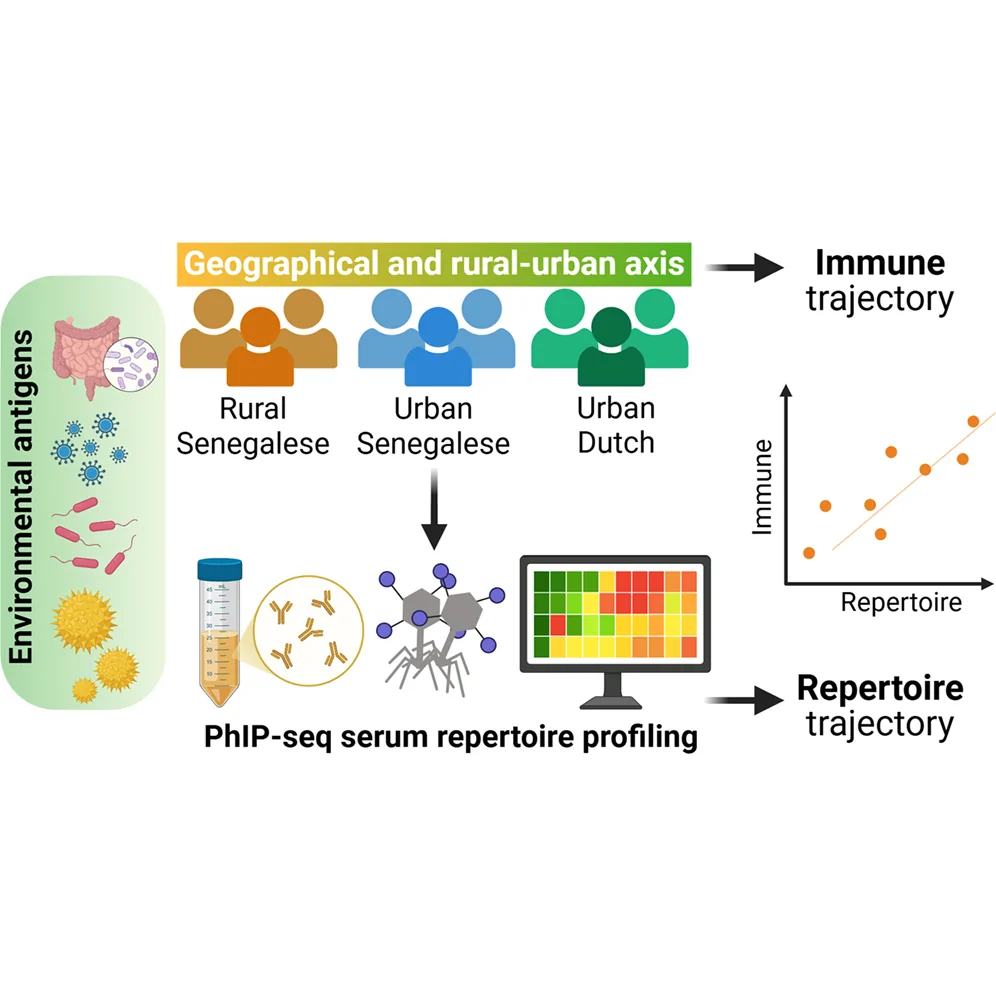

## Teaser

Cell biology; Immunology; Microbiology; Virology

## Introduction

Populations living along rural-to-urban gradients exhibit marked differences in immune profiles,^1^^,^^2^^,^^3^^,^^4^ disease susceptibility, and vaccine responses,^5^ yet the specific environmental exposures driving these differences remain poorly characterized. Emerging evidence reveals that gut microbiome composition and lifestyle factors vary across rural-urban gradients and correlate with immune phenotypes. Rural Tanzanians have a higher microbial diversity and enrichment of *Prevotella* compared to their urban counterparts, reflecting broader differences between low- and middle-income countries (LMICs) versus high-income countries (HICs).^6^ Lifestyle scores (LSs) also correlate with immune phenotypes, as rural-associated LSs predict more activated immune profiles than urban-associated LSs.^7^

Specific microbial exposures—malaria, *Mycobacterium*, helminths, fungi, and cytomegalovirus (CMV)—can profoundly shape immune profiles through diverse mechanisms in both LMIC and HIC settings. Repeated malaria infections drive distinct transcriptomic profiles in immune cells of Kenyan children,^8^ while *Mycobacterium* exposure—both through Bacillus Calmette–Guérin (BCG) vaccination and environmental encounters—remodels innate immune responses.^9^ Fungal β-glucan exposure enhances monocyte responses to unrelated pathogens,^10^ and helminth infections selectively expand type 2 and regulatory T and B cell populations.^3^ CMV-seropositive individuals in HICs exhibit reduced vaccine responses against Ebola compared to their seronegative counterparts, in exposed versus naive British adults.^7^ These findings illustrate how cumulative microbial exposures shape population-specific immune landscapes, yet systematic approaches linking exposure histories with immune phenotypes remain limited.

Conventional methods cannot assess exposure breadth at scale. ELISAs and peptide arrays allow screening for only 100s to 1,000s of antigens,^11^^,^^12^ while viral and bacterial pathogens, commensals, and environmental antigens represent an antigen space that is several orders of magnitude larger. Phage-displayed immunoprecipitation sequencing (PhIP-seq) enables scalable, high-resolution profiling of individual antibody repertoires against 100,000s of epitopes as molecular records of microbial encounters.^13^^,^^14^^,^^15^ Prior work demonstrated its value in autoimmunity,^16^^,^^17^^,^^18^ microbiome-directed responses,^19^ antibody responses against dietary and environmental antigens,^20^ and population-level antibody diversity in northern Netherlands^21^—but cross-population comparisons are unrealized.

Here, we used PhIP-seq to profile antibody repertoires across individuals residing in rural Senegal, urban Senegal, and urban Netherlands, revealing population-specific antibody patterns along a rural-urban trajectory that correlate with baseline immune signatures. This establishes a framework for understanding how environmental exposure histories shape immune landscapes in understudied populations.

## Results

### Study population

**Figure 1:**
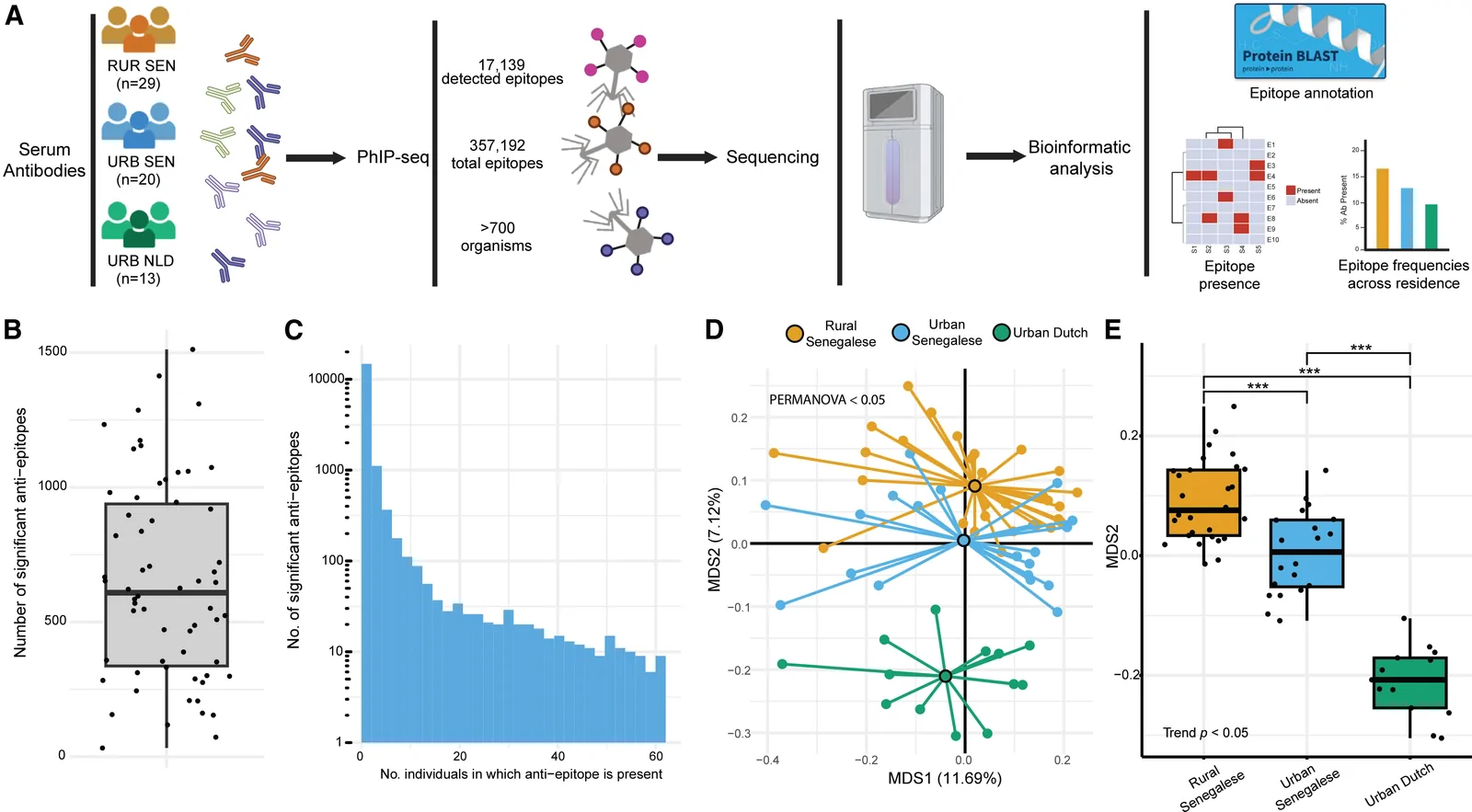
PhIP-seq profiling reveals ordered antibody repertoire variation across residence groups (A) Experimental overview. Sera from rural Senegal (*n* = 29), urban Senegal (*n* = 20), and urban Dutch (*n* = 13) participants were incubated with a peptide-phage library containing 357,192 epitopes pooled from three previously reported libraries.^19^^,^^20^^,^^22^ Immunoprecipitation was used to capture antibody-bound phages, which were subsequently sequenced and annotated using protein BLAST to characterize epitope targets and extract antibody repertoire patterns across groups. Created using BioRender.com. (B) Number of significant anti-epitopes per individual. Boxplots show median, quartiles, and whiskers extending 1.5× the interquartile range. See Figure S1 for comparisons between residence areas. (C) Distribution of anti-epitope detection frequency across individuals. *y* axis shows log10-transformed number of anti-epitopes. (D) Multidimensional scaling (MDS) plot of the filtered PhIP-seq data using the Jaccard distance metric. Individuals are colored by residence group; large circles represent group centroids (the mean MDS1 and MDS2 coordinates of each residence group). PERMANOVA *p* value is shown. (E) Boxplot showing MDS2 values by residence group. *p* values from linear trend test and Tukey post hoc pairwise comparisons are indicated. ∗∗∗*p* < 0.001.

To investigate antibody repertoire profiles along the rural-urban axis, we analyzed 62 age-matched healthy adults from rural Senegal (*n* = 29), urban Senegal (*n* = 20), and urban Netherlands (*n* = 13) (Figure 1A). Participants were matched for age (median 27 years, range: 18–43 years; Kruskal-Wallis *p* = 0.55) but not sex. Sex distribution was balanced within the Senegalese cohorts (rural: 14/29 female, 48.3%; urban: 10/20 female, 50.0%) but skewed toward females in the Dutch cohort (11/13 female, 84.6%; chi-square *p* = 0.069). All Senegalese participants had maintained residence in their respective environments for ≥10 years. As described previously,^23^ rural Senegalese participants exhibited typical rural lifestyle features, including larger household sizes and traditional housing with earth floors and mud walls compared to their urban counterparts.

### Antibody repertoires vary across rural and urban populations

PhIP-seq was performed on all 62 samples against a library of 357k peptide antigens,^19^^,^^20^^,^^22^ probing more than 20 million serum-antigen interactions. Across all individuals, antibodies were detected against 17,139 epitopes derived from over 700 organisms (Figure 1A and Data S1). Individual antibody repertoires contained a median of 609 significant anti-epitope responses (Figure 1B), with marked heterogeneity in epitope recognition across individuals: 12,647 epitopes were detected in at most one individual, whereas 60 epitopes were recognized by more than 50 (>80%) participants (Figure 1C). The total anti-epitope numbers did not differ significantly between residence groups (Figure S1).

Multidimensional scaling (MDS) revealed significant differences in overall antibody repertoire patterns between the Dutch and rural Senegalese populations (PERMANOVA *p* < 0.001), with individuals ordered sequentially along the MDS2 axis according to their residence group (Figures 1D and 1E). This ordering, from rural Senegalese through urban Senegalese to urban Dutch, is consistent with progressive differences in cumulative microbial exposure. To test the robustness of this ordering, we repeated the MDS using three distance metrics (Bray-Curtis, Euclidean, and Jaccard) and four filtering thresholds. MDS1 (non-diagonal Pearson r: median = 0.97, min = 0.61, and max = 0.99) and MDS2 (non-diagonal Pearson r: median = 0.94, min = 0.82, and max = 0.99) were highly concordant across all settings (Figure S2).

The total number of significant anti-epitopes detected per individual significantly correlated with MDS1 but not with MDS2 (Figures S3A–S3C). Age showed no significant association with antibody repertoire composition (Figure S3D). A significant difference between sexes was observed across the full cohort (Figure S3E). Because the Dutch cohort is predominantly female and contributes substantially to the Senegal-versus-Netherlands contrast, we could not separate sex from geography by excluding the Dutch. Instead, to assess sensitivity to sex ratio while retaining the Dutch group, we down-sampled the Dutch females to three (keeping the two males) and reran the MDS. A significant trend across MDS2 was still observed, and the groups remained significantly different across the MDS1-MDS2 space (Figures S3G and S3H). While this indicates that the rural-urban signal is not solely an artifact of sex imbalance, sex and geography remain partially confounded in the Senegal-versus-Netherlands comparison.

### Pathogen-specific antibody signatures are structured by geography and rural-urban residence

**Figure 2:**
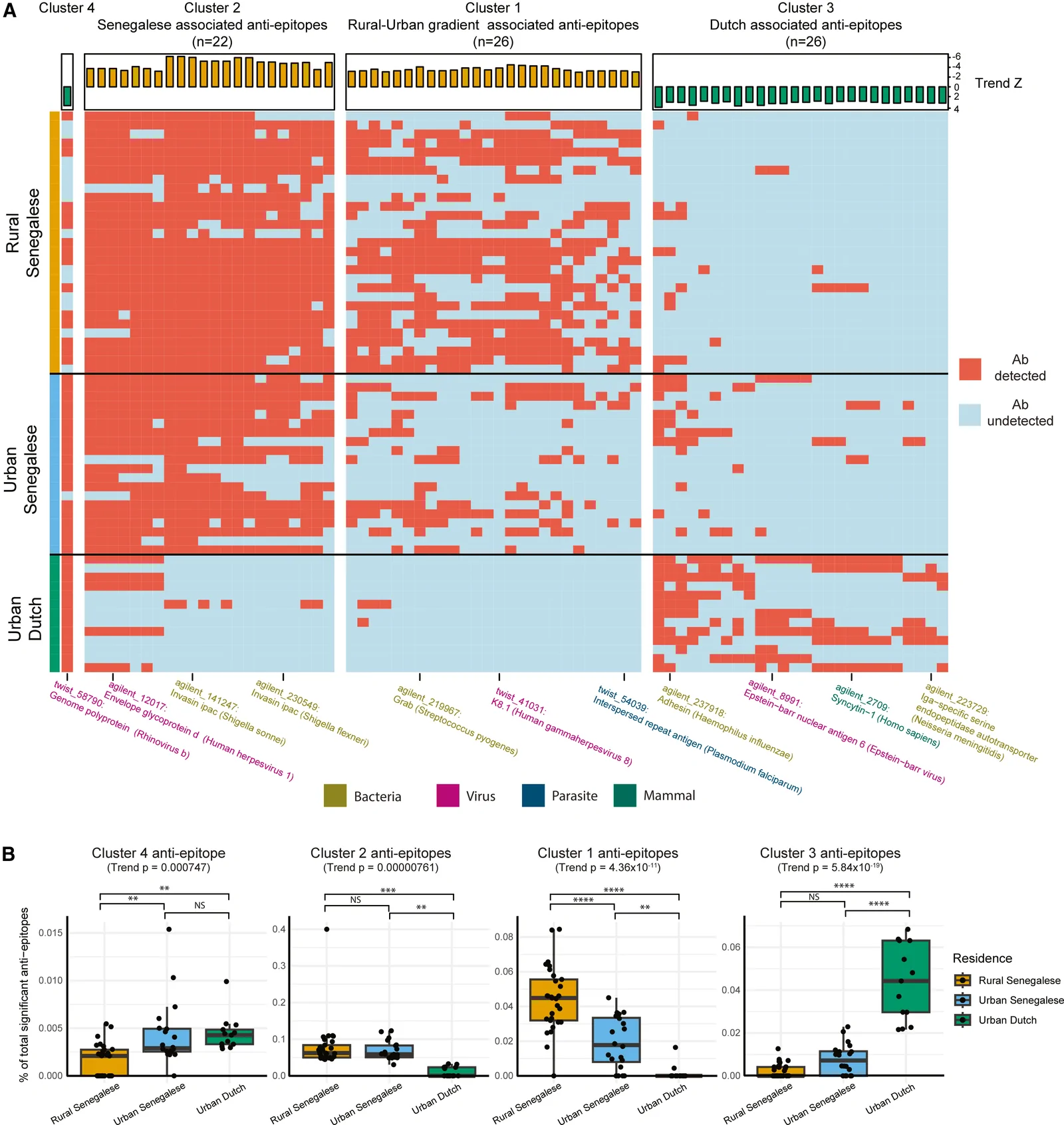
Antibody repertoires cluster into geographically restricted and ordered rural-urban axis signatures (A) Clustered heatmap showing whether antibody (Ab) against epitopes (columns) was detected (red) or undetected (blue) across individuals (rows). The heatmap is organized by residence group (row-wise) and *k*-means-clustered epitopes (column-wise). Top barplots show the Z statistic of the Cochran-Armitage trend test. Selected epitopes are annotated and color-coded by taxa. (B) Cluster module scores by residence group. Each individual’s module score is the proportion of a cluster’s anti-epitopes (from A) detected in that individual. Boxplots show the median (center line), interquartile range (box limits), and 1.5× the interquartile range (whiskers), with individual data points overlaid (rural Senegalese, *n* = 29; urban Senegalese, *n* = 20; urban Dutch, *n* = 13). Linear trend and pairwise test *p* values are shown. NS, *p*/FDR ≥ 0.05; ∗*p*/FDR < 0.05; ∗∗*p*/FDR < 0.01; ∗∗∗*p*/FDR < 0.001.

Hierarchical clustering of antibody repertoire profiles revealed distinct exposure history signatures across residence groups (Figures 2A and S4). To quantify these cohort-stratified patterns, we summarized each individual’s reactivity toward a cluster as a module score, defined as the proportion of that cluster’s anti-epitopes detected in the individual (Figure 2B). Cluster 1 scores (26 anti-epitopes) followed a rural-urban axis, highest in rural Senegalese, intermediate in urban Senegalese, and lowest in urban Dutch individuals (trend *p* = 4.36e−11). Clusters 2 and 3 contained geographically restricted signatures. Cluster 2 scores (22 anti-epitopes) were elevated in both Senegalese groups relative to the Dutch but did not differ between rural and urban Senegalese (*p* = 0.723). Cluster 3 scores (26 anti-epitopes) were elevated in urban Dutch relative to both Senegalese groups and did not differ between rural and urban Senegalese. These cluster-level scores show that antibody reactivity is structured both geographically and along the rural-urban axis. To resolve which individual anti-epitopes drive these patterns, we next examined the gradient at single-epitope and organism resolution.

**Figure 3:**
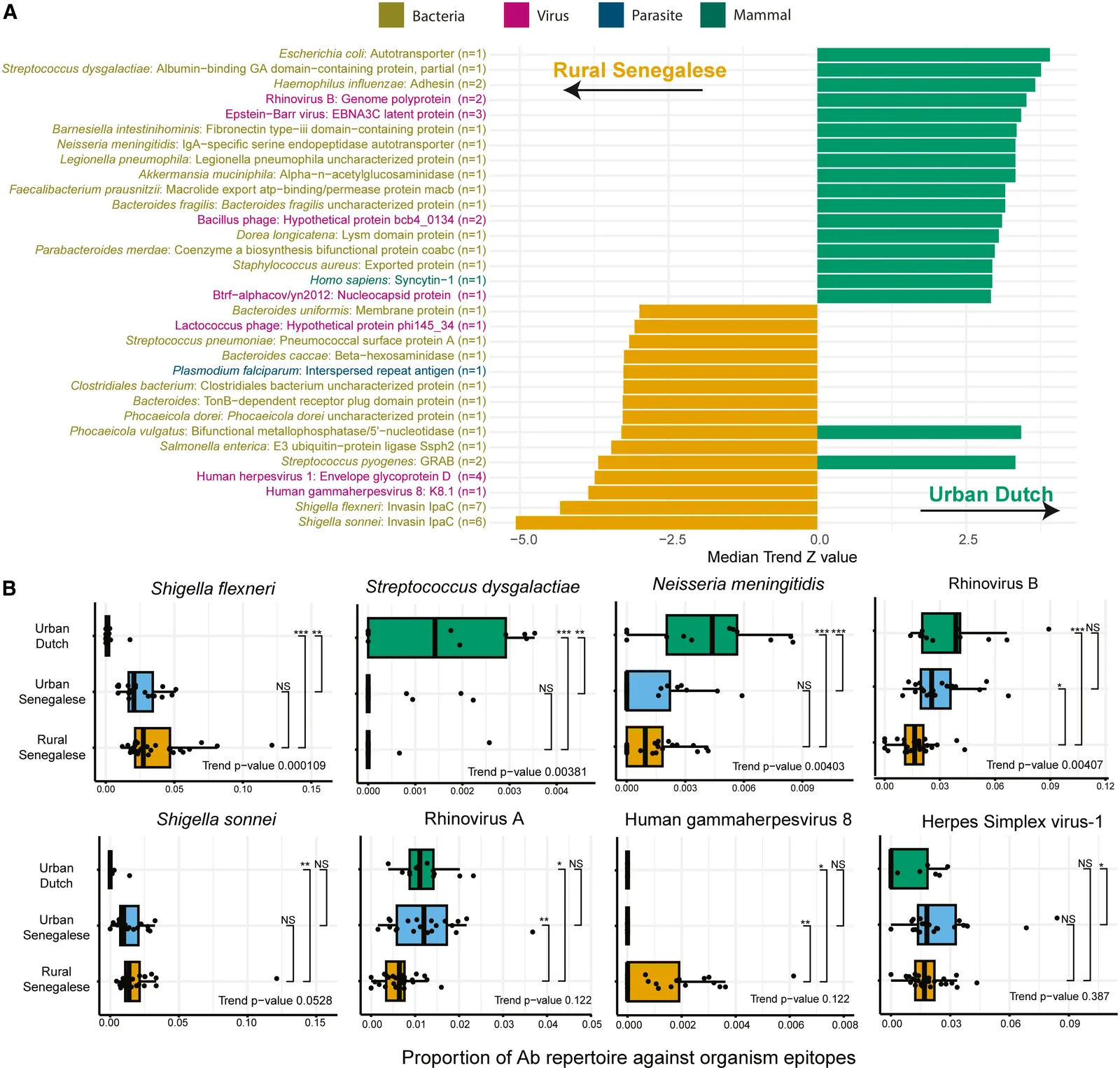
Pathogen-specific antibody responses trend across the rural-urban axis (A) Median trend-test Z-values for anti-epitopes showing significant linear trends across the rural-urban axis. Cochran-Armitage trend tests were performed per epitope and results, with results aggregated by organism and direction of association. For each organism, the most common protein annotation and number of significant anti-epitopes (*n*) are displayed. Organisms are color-coded by taxa. (B) Proportion of individual antibody repertoire targeting epitopes from selected organisms by residence group. Boxplots show median, quartiles, and whiskers extending 1.5× the interquartile range. Linear trend and pairwise test *p* values are shown (FDR-corrected). NS, *p*/FDR ≥ 0.05, ∗*p*/FDR < 0.05, ∗∗*p*/FDR < 0.01, and ∗∗∗*p*/FDR < 0.001.

We performed linear trend analysis across the three residence groups in the order rural Senegalese, urban Senegalese, and urban Dutch, testing each anti-epitope for an ordered linear trend in detection frequency along this gradient. Of 75 anti-epitopes showing significant gradient associations, 27 increased in frequency from rural Senegal to urban Netherlands and 48 decreased (Figures 3A, S5, S6; Data S2). As a robustness check of our statistical test, we repeated the analysis with two alternative orderings that move urban Senegalese out of the intermediate position: first, urban Senegalese, rural Senegalese, and urban Dutch; and second, urban Senegalese, urban Dutch, and rural Senegalese. The first returned 8 significant anti-epitopes, 4 against *Shigella flexneri*, 3 against *Shigella sonnei*, and 1 against HHV-1, all among the original 75. The second ordering returned no significant results. Together, these findings indicate that antibody repertoire signatures are structured across both the rural-urban axis and geographic regions, consistent with differences in microbial exposure between these settings.

Pathogen-specific antibody patterns varied systematically across the rural-urban axis. Rural Senegalese individuals showed the highest detection rates for epitopes from *Shigella* spp., herpes simplex virus, and CMV, while urban Dutch individuals showed the highest detection for rhinovirus B, Epstein-Barr virus (EBV), and *Haemophilus influenzae* (Figure 3A). Many trending anti-epitopes were geographically restricted: 32 of the 48 trending toward rural Senegal were undetected in any Dutch individual, and 12 of the 27 anti-epitopes trending toward urban Netherlands were absent from Senegalese repertoires (Figures S5 and S6). The cohort also shared universal responses; for example, *Streptococcus pneumoniae* pneumococcal histidine triad protein epitopes were recognized by 61 of 62 individuals (Data S1). Two of the five most widely detected epitopes across the cohort derive from *S. aureus* staphylococcal protein A, recognized in 61/62 individuals regardless of residence. As staphylococcal protein A is known to bind IgG non-specifically through its Fc region, this finding is not unexpected.^19^

Since individual epitopes from the same pathogen sometimes showed divergent trends along the rural-urban axis, we assessed pathogen-level enrichment by calculating the aggregated proportion of each individual’s repertoire targeting organisms with significant linear associations (Data S2). Analysis of the four organisms with the lowest adjusted *p* values for each direction of association confirmed the epitope-level findings: *Shigella sonnei*, *S. flexneri*, and herpes simplex virus 1 showed significant rural-trending responses, while rhinovirus B, *Neisseria meningitidis*, and *Streptococcus dysgalactiae* trended toward urban Dutch subjects (Figure 3B). Geographic restriction in anti-epitope detection was particularly striking—nearly all Senegalese individuals (48/49) recognized *Shigella sonnei* epitopes compared to only three Dutch participants. Responses against human gammaherpesvirus 8 (Kaposi’s sarcoma-associated herpesvirus [KSHV]) were detected exclusively in rural Senegalese individuals (14/29) and were completely absent from urban Senegalese and Dutch groups (Figure 3B). We also tested each anti-epitope for a quadratic trend, which would identify anti-epitopes highest or lowest in the urban Senegalese relative to the two endpoint groups and so argue against an ordered axis. No anti-epitope reached significance for a quadratic trend, indicating that the significant linear signal is not driven by a non-monotonic pattern in which the urban Senegalese diverge from both rural Senegalese and urban Dutch.

As antibody responses against certain organisms were exclusive to particular residence groups, we next assessed organism-level diversity of antibody repertoires across the gradient. There was no significant linear trend in expected richness or Simpson’s index across the rural-urban axis, although rural Senegalese individuals showed higher overall diversity compared to urban Senegalese and Dutch participants (Figures S7A and S7B).

### PhIP-seq anti-epitope trajectory correlates with rural-urban immune signatures

**Figure 4:**
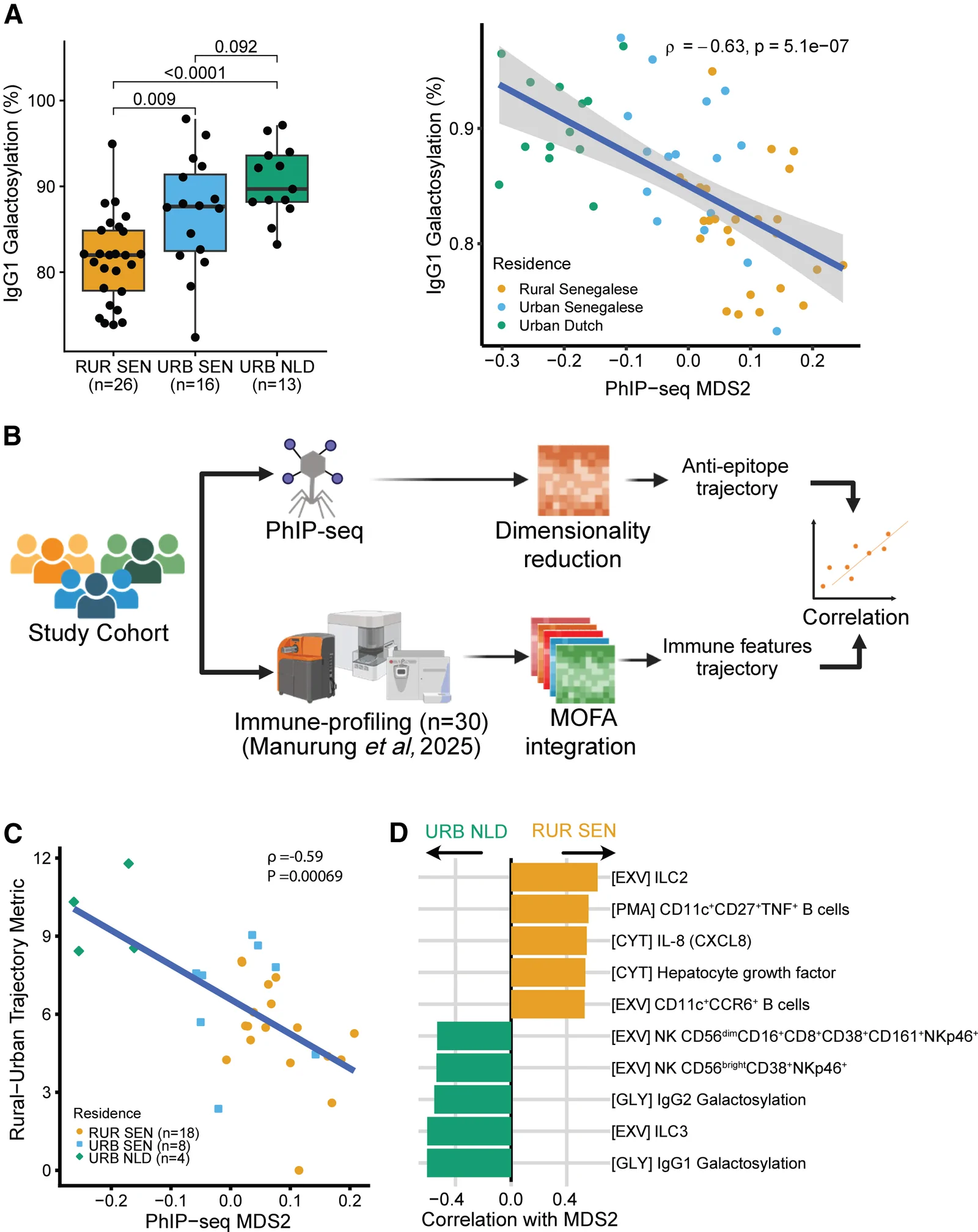
The antibody repertoire trajectory correlates with rural-urban immune signatures (A) IgG1 galactosylation levels by residence (left; pairwise *t* test *p* values shown) and correlation with PhIP-seq MDS2 (right; Spearman’s rho [ρ] and *p* value shown). (B) Analysis scheme for integrating anti-epitope trajectory obtained from PhIP-seq (PhIP-seq MDS2) with the previously published immune feature trajectory (rural-urban lambda metric). Created using Biorender.com. (C) Correlation between PhIP-seq MDS2 scores and rural-urban lambda metric. Spearman’s rho and *p* value are shown with fitted regression line. (D) Immune features significantly correlated with PhIP-seq MDS2 (FDR < 0.1), ranked by correlation coefficient magnitude and direction.

Having established distinct antibody profiles across residence groups, we investigated whether these exposure signatures correlate with immune activation. IgG1 Fc galactosylation is an established marker of immune activation that varies with environmental settings. Lower galactosylation is associated with rural residence, parasitic infection, and elevated C-reactive protein, whereas higher galactosylation characterizes urban communities and more affluent countries.^23^^,^^24^ This marker, which was available for 26/29 rural Senegalese individuals, 16/20 urban Senegalese individuals, and all the urban Dutch individuals, showed a clear ordered pattern across residence groups: lowest in rural Senegalese, intermediate in urban Senegalese, and highest in urban Dutch participants (Figure 4A). Moreover, IgG1 galactosylation levels showed a strong negative correlation with PhIP-seq PC2 scores (Spearman’s ρ = −0.64, *p* < 0.0001), suggesting that population-specific microbial exposure histories are reflected in systemic immune activation status.

We next integrated PhIP-seq data with comprehensive immune profiling data from the same individuals (Figure 4B; *n* = 18 rural Senegalese, 8 urban Senegalese, and 4 urban Dutch). These profiling data, previously described in detail,^23^ comprise five complementary datasets: high-dimensional mass cytometry-based immune cell phenotyping of peripheral blood (*ex vivo*; EXV) and after stimulation with PMA (phorbol 12-myristate 13-acetate)/ionomycin or monophosphoryl lipid-A (MPL), IgG Fc glycan profiling by liquid chromatography-mass spectrometry (LC-MS) (GLY), and serum cytokine quantification by Luminex (CYT). Our previously established rural-urban immune trajectory lambda metric^23^—derived from unsupervised multi-dataset integration of these modalities—correlated significantly with PhIP-seq PC2 scores (Spearman’s ρ = 0.55, *p* = 0.0021; Figures 4C and S8A), demonstrating convergence between antibody-defined microbial exposure history and broader systemic immune phenotypes.

To identify immune features driving this association, we correlated PhIP-seq MDS2 scores with all immunological parameters from our published dataset. Ten immune features exhibited significant associations at false discovery rate (FDR) <0.1 (Figue S8B), revealing distinct immunological signatures linked to environmental exposure patterns. Rural residence-associated antibody profiles correlated with higher frequencies of type 2 innate lymphoid cells (ILC2s), CD11c^+^ B cells, as well as elevated serum IL-8 and hepatocyte growth factor (HGF) levels (Figures 4D and S8C). Conversely, urban-associated profiles correlated with increased ILC3 and NKp46^+^ natural killer (NK) cell frequencies alongside higher IgG1/2 Fc galactosylation. These findings show that serum antibody repertoires serve as a correlate for microbial exposure histories and may reflect downstream variation in immune phenotype.

Trajectory-level correlation cannot reveal which individual anti-epitopes and immune features co-vary. We therefore integrated the two modalities using two-way orthogonal partial least squares (O2PLS),^25^ a multivariate analysis method that models their shared variation for an exploratory, hypothesis-generating analysis, considering our limited sample size. Across the 30 individuals with paired data, the first joint component ordered individuals by residence along the rural-urban axis (Figure S9), recapitulating the trajectory-level convergence. At the rural end of this component, the highest-loading PhIP-seq features were antibody responses against *Shigella flexneri* and *S. sonnei*, matching the organism-level enrichments in Figure 3B; at the urban Dutch end, they were responses against *Bacteroidales* and bacteriophage epitopes. The immune features split the same way: CD11c^+^ B cells and effector/memory T cells loaded toward the rural end and IFNγ-producing NK and γδ T cells toward the urban end (Figure S9). These loadings agree with the aforementioned trajectory-level associations and with the rural-urban immune signatures we reported previously.^23^

## Discussion

Using PhIP-seq, we profiled antibody repertoires of individuals across rural and urban populations and revealed distinct antigen exposure histories that correlate with environmental and lifestyle factors. Repertoires of rural Senegalese individuals were enriched for antibodies against pathogens associated with poor sanitation and endemic exposure, including *Shigella* species, human herpesviruses, and *Plasmodium falciparum*. Conversely, urban Dutch repertoires were enriched for antibodies against common respiratory pathogens and commensals, including rhinovirus B, EBV, and *Haemophilus influenzae*. These patterns align with known epidemiological differences in infectious disease burden between LMICs and HICs.

Interestingly, antibodies against human gammaherpesvirus 8—also known as KSHV—were detected exclusively among rural Senegalese participants and were completely absent from both urban Senegalese and Dutch groups. KSHV is known to be highly prevalent in sub-Saharan Africa, and its higher prevalence in rural regions has been described previously.^26^ Studies have linked higher KSHV prevalence to factors typically associated with rural living, including denser households, which facilitate viral transmission,^27^ and *Schistosoma* infections, which induce viral reactivation^28^—factors also present in rural Senegal. The higher percentage of individuals with antibodies against KSHV in our Senegalese compared to Dutch populations mirrors previous findings in Brazilian Amerindians.^29^ Furthermore, the higher prevalence of antibodies against rhinovirus B in the Dutch population compared to the Senegalese parallels findings from a US-based cohort. These results demonstrate commonality in exposure patterns across different LMICs and HICs, respectively, and highlight the need to compare rural and urban areas across multiple LMICs to identify further similarities or differences in exposure.

It is notable that antibodies against certain epitopes are enriched in particular populations and that antibodies against epitopes from the same organism can display opposite enrichments. Enrichment of antibodies against particular epitopes has been reported in the context of disease, with antibodies against EBV lytic-phase proteins significantly enriched in individuals with primary sclerosing cholangitis compared to controls^30^ and antibodies against bacterial flagellin epitopes enriched in individuals with Crohn’s disease.^16^ In our study, the antibody response to *S. aureus* stands out, with antibodies simultaneously detected in most individuals for some epitopes while others are enriched toward opposite ends of the rural-urban axis. Notably, two of the five most widely detected epitopes across the cohort derive from *S. aureus* staphylococcal protein A, which is known to bind IgG non-specifically through its Fc region,^19^ suggesting that these near-universal responses reflect non-specific antibody capture rather than genuine exposure-driven immunity. Taken together, the implications of epitope-specific enrichment patterns, especially for vaccine development, warrant further investigation.

Differences in immune profiles across the rural-urban axis are hypothesized to be driven, at least in part, by pathogen exposure in the environment,^23^^,^^24^ as suggested by differences in infectious disease burden between LMICs and HICs.^31^^,^^32^ Here, we demonstrated convergence between independently constructed trajectories of antibody repertoire profiles and immune remodeling along the rural-urban axis. This analysis further revealed specific immune features associated with rural-urban exposure histories. For example, ILC2s, which were associated with rural repertoire profiles, are known to be expanded following helminth infections.^3^ Rural-associated profiles also correlated with elevated serum IL-8 and HGF levels, consistent with heightened innate immune activation. Conversely, urban repertoire profiles were associated with higher frequencies of NK cells expressing NKp46, a critical marker for controlling viruses such as influenza,^33^ as well as increased ILC3 frequencies and higher IgG1 and IgG2 Fc galactosylation—a biomarker of reduced immune activation.^22^ Future studies with larger sample sizes will provide greater statistical power to link specific microbial epitopes to immune parameters and to further our understanding of the mechanistic links between exposures and immune remodeling.

These findings highlight the need to utilize this technology to further investigate the link between microbial exposure and immune profile. This investigation can be expanded in multiple ways. The first would be to increase sample size to numbers comparable to other PhIP-seq studies,^16^^,^^21^ allowing greater statistical rigor and overcoming interpretation issues confounded by individual library size, such as exposure diversity and read count levels. The second expansion would be in the pathogen epitopes investigated. Most epitopes in the current libraries derive from microbiome organisms, reflecting the origins of PhIP-seq in studying inflammatory bowel diseases.^16^^,^^19^ When assessing differences across the rural-urban axis, it would be beneficial to include epitopes from pathogens common to LMICs but not HICs, such as helminths.^32^ Nevertheless, the fact that most of our findings align with epidemiological observations—such as higher detection of KSHV in rural Senegal and *Shigella* enrichment along the sanitation gradient—highlights the strength of PhIP-seq as a tool for broad, systematic surveys of microbial exposures and may inform regional disease screening policies.

In conclusion, PhIP-seq robustly captures differential microbial exposure patterns across populations that are either geographically restricted or vary across rural and urban populations, consistent with our previous immune profiling study, providing insights into how environmental exposures shape population-specific immune signatures. This approach establishes a framework for understanding immune adaptation in diverse global populations and environments.

### Limitations of the study

Several limitations should be considered. The three residence groups differ on many co-varying factors beyond urbanization, including ancestry, diet, healthcare access, vaccination and infection histories, sanitation, and microbiome composition. The ordered antibody signatures we describe should therefore be read as exposure-associated variation across populations rather than as effects of urbanization specifically. Next, microbial taxonomic assignment was based on sequence similarity to reference databases. For short peptides, particularly those from conserved or homologous protein domains, the top hit may not reflect the true organism of origin, and cross-reactivity is plausible, as may be the case for the *Shigella* and rhinovirus results. Organism-level signals should therefore be interpreted as putative taxonomic or functional indicators rather than evidence of exposure to a specific microorganism, and given more weight when supported by multiple peptides or shared protein annotations rather than single hits. Furthermore, the library is restricted to peptides of 54 or 64 amino acids and so does not capture conformational epitopes dependent on native protein folding. However, these peptides are considerably longer than minimal linear epitopes (∼5–20 amino acids) and may form local structural elements such as α helices or β strands that contribute to antibody recognition. PhIP-seq thus provides a broad readout of linear and locally structured antigenic domains and at the scale of this library (∼357,000 antigens) covers a far wider antigenic space than targeted approaches such as ELISA or peptide arrays. Finally, although the Senegalese cohorts were sex balanced, the Dutch cohort was not, leaving sex and geography partially confounded in the Senegal-versus-Netherlands contrast. Differences between the sex-balanced rural and urban Senegalese groups were nonetheless observed (Figures 2B and 3B), indicating that the rural-urban repertoire signal does not depend solely on the Dutch group.

## Resource availability

### Lead contact

Requests for further information and resources should be directed to and will be fulfilled by the lead contact, Mikhael D. Manurung (m.d.manurung@lumc.nl).

### Materials availability

This study did not generate new unique reagents.

### Data and code availability

• Processed immunoprofiling data for a subset of individuals and the corresponding analysis scripts have been made publicly available in Zenodo: https://doi.org/10.5281/zenodo.14967877. Raw data are available upon reasonable request to the authors.
• The code used to generate the results can be found at the following GitHub page: https://github.com/No2Ross/urbanization-antibody-landscapes/.
• Any additional information required to reanalyze the data reported in this paper is available from the lead contact upon request.

## Acknowledgments

We thank the laboratory members of the M.Y. group for their critical feedback. We would like to acknowledge all staff members in Senegal (field workers and medical staff of the Health Centre of Richard Toll and staff of the Immunology Laboratory at Cheikh Anta Diop University, Senegal) and the Netherlands who helped to make this study possible. Last, we would like to thank all volunteers who participated in this study, without whom the study could not have been performed. This study is part of the EDCTP2 program supported by the 10.13039/501100000780European Union (grant no. TMA20216CDF-1595). M.Y. was supported by an NWO Spinoza Prize 2021, an NWO -WOTRO Science for Global Development Programme (grant no. W 07.30318.019), and an ERC Advanced Grant (grant no. 101055179). M.D.M. was supported by the 10.13039/100000923Silicon Valley Community Foundation (DAF2020-217471). T.V. is supported by an ERC Starting Grant (EarlyMicroAbs) and coordinates the EU Horizon Health consortium grant ID-DarkMatter-NCD (grant no. 101136582).

## Author contributions

M.Y., M.M., and M.D.M. were responsible for the study design. R.F.L. and M.D.M. were responsible for the statistical analysis and data interpretation and prepared the first draft. M.Y., M.M., and M.D.M. acquired funding. G.I. and T.V. generated the data and optimized the PhIP-seq experimental protocols. All authors reviewed the manuscript.

## Declaration of interests

The authors declare that they have no competing interests. The funding sources had no role in collecting, analyzing, interpreting, or reporting the data. The opinions expressed in this document reflect only the author’s view. The funding sources are not responsible for any use that may be made of the information that it contains.

## STAR★Methods

### Key resources table

**Table:** 

REAGENT or RESOURCE	SOURCE	IDENTIFIER
**Antibodies**		

HRP conjugated anti human IgG antibody	Southern Biotech	Cat#204205
Mouse anti-human IgG Fc-BIOT	Southern Biotech	Cat#9040-08

**Bacterial and virus strains**		

T7Select 10-3 cloning kit	Merck	Cat#70550-3

**Biological samples**		

62 serum blood samples, 29 from rural Senegal, 20 from urban Senegal and 13 from urban Netherlands	Manurung et al.^23^	https://doi.org/10.1126/sciadv.adu0419

**Chemicals, peptides, and recombinant proteins**		

IPEGAL CA 630	Sigma-Aldrich	Cat#I3021
Protein A magnetic beads	Thermo Fisher Scientific	Cat#10008D
Protein G magnetic beads	Thermo Fisher Scientific	Cat#10009D
Thermo Scientific™ 1-Step™ TMB ELISA Substrate Solutions	Thermo Fisher Scientific	Cat#34022
Q5 polymerase	New England Biolabs	Cat#M0493L
Bovine Serum Albumin, heat shock fraction, pH 7, ≥98%	Sigma-Aldrich/Merck	Cat#A7906-100G

**Critical commercial assays**		

QIAquick gel extraction kit	Qiagen	Cat#28704
QIAquick PCR purification kit	Qiagen	Cat#28104

**Deposited data**		

Raw data for PhIP-seq experiments on rural and urban Senegalese and urban Dutch	This paper	TBD
Processed data for multi-omic experiments on rural and urban Senegalese and urban Dutch	Manurung et al.^23^	https://zenodo.org/records/14967877

**Oligonucleotides**		

Library amplification primer fwd	GATGCGCCGTGGGAATTCT	N/A
Library amplification primer rev	GTCGGGTGGCAAGCTTTCA	N/A

**Recombinant DNA**		

Oligo pool (200 mers)	Twist Bioscience	N/A
Oligo pool (230 mers)	Agilent Technologies	N/A

**Illumina amplicon sequencing primers**		

PCR1	tcgtcggcagcgtcagatgtgtataagagacag GTTACTCGAGTGCGGCCGCAAGC	N/A
	gtctcgtgggctcggagatgtgtataagagacag ATGCTCGGGGATCCGAATTC	N/A
PCR2	Illumina Nextera 10-nt unique dual index primers	N/A
PCR3	AATGATACGGCGACCACCGA	N/A
	CAAGCAGAAGACGGCATACGA	N/A
R1 primer	ttactcgagtgcggccgcaagctttca	N/A
R2 primer	tgtgtataagagacagatgctcggggatccgaattct	N/A

**Software and algorithms**		

Peptide quantification and enrichment determination	Vogl et al.^19^ and Leviatan et al.^20^^,^^34^	N/A
Descriptive statistics, association analyses, prediction analyses	This paper	https://github.com/No2Ross/urbanization-antibody-landscapes
R (4.5.2)	The R foundation	https://www.r-project.org
emmeans (2.0.3)	The Comprehensive R Archive Network	https://CRAN.R-project.org/package=emmeans

**Other**		

Nunc™ Immobilizer™	Thermo Scientific	Cat#436014
BioTides™ Peptides	JPT Peptide Technologies (Berlin, Germany)	N/A
agilent_23359	RTGVPASTWAAIIARESNGQ	N/A
twist_95165	SSTWSTIINRESKGNVNISN	N/A
agilent_90413	GLSISEKMRSQIRGLNKASS	N/A
twist_58059	SMAITFKRALGARPKQPPPR	N/A
aglient_128883	GLSISEKMRSQIRGLNKASS	N/A
agilent_131884	GLTISEKMRKQIKGLDQAST	N/A
agilent_12020 (positive control)	GRRPFFHPVAEADYFEYHQE	N/A
agilent_1576 (negative control)	KIMGMEFSEEKIQYLVYQML	N/A
Fluent 1080 liquid handling robot	Tecan	N/A
MASTERBLOCK, 96w, PP, 2 mL, Natural, 50/case	Danyel biotech	Cat#60-780270
Corning Axygen® AM-2 ML-SQ AxyMat™	Biolab Ltd	Cat#AXY-AM-2 ML-SQ

### Experimental model and study participant details

#### Human study cohort

Participants were recruited between November 2018 and August 2019 from three sites: rural Senegal (Pakh and Richard Toll; *n* = 72), urban Senegal (Dakar; *n* = 46), and the Netherlands (Leiden; *n* = 28). Recruitment was coordinated through local healthcare centers in Senegal and Leiden University Medical Center. Written informed consent was obtained from all participants. The study was approved by the ethics committees of Cheikh Anta Diop University of Dakar (0339/2018/CER/UCAD) and Leiden University Medical Center (NL66287.058.18, NL-OMON48968).

Rural Senegalese participants originated from agricultural villages in the Senegal River basin, where schistosomiasis has historically been highly endemic. Urban Senegalese participants were recruited from Dakar, a non-endemic area with improved sanitation and consistent access to clean water. Retrospective surveys in Dakar reported intestinal parasite prevalence of ∼19–26%, although estimates may be biased by sampling from symptomatic patients. Rural and urban classifications were confirmed using questionnaire data on household size, animal contact, housing conditions, and amenities. The Senegalese population is ethnically diverse (predominantly Wolof, Pular, and Serer), though cultural admixture is common; most participants were Wolof.

Inclusion criteria were: age 18–40 years, residence in the study area ≥10 years, and absence of chronic illnesses (e.g., diabetes, hypertension). Participants underwent clinical examination and completed standardized questionnaires covering demographic, socioeconomic, lifestyle, and medical history information. Exclusion criteria were evidence of infection with *Plasmodium* spp. (thick smear, rapid diagnostic test), *Schistosoma* spp. (Kato-Katz and urine filtration, 12 μm filters), or intestinal helminths (*Ascaris lumbricoides*, *Trichuris trichiura*, hookworm) as determined by stool microscopy.

To ensure comparable sample quality across sites, technical staff received centralized training at Leiden University Medical Center and adhered to standardized operating procedures (SOPs) for sample collection and processing. Venous blood was drawn into heparin, EDTA, and dry tubes for cell isolation, full blood count, and serology testing.

#### Description of immune-profiling datasets

Five datasets from our previously published study^23^ were re-analyzed in this manuscript: peripheral blood immune cell phenotyping (*ex vivo*; EXV), cytokine production following 6-h stimulation with PMA/ionomycin or monophosphoryl lipid-A (MPL), IgG Fc glycan profiling (GLY), and serum cytokine levels (CYT). Detailed methodological description for the acquisition, pre-processing, and analysis for each dataset as well as the integrative analysis of these datasets can be found elsewhere.^23^

Briefly, the EXV, PMA, and MPL datasets were obtained from mass cytometry analysis of peripheral blood mononuclear cells (PBMCs). Cryopreserved PBMCs (3×10^6^ cells) were cultured with either PMA/ionomycin or MPL for 6 h (Brefeldin A added for the final 4 h), then stained using two standardized antibody panels for surface markers, intracellular cytokines, and nuclear antigens, with dead cell discrimination via intercalator staining. Cells were acquired on a Helios mass cytometer with automated tuning, normalization using calibration beads, and quality control through FlowJo gating and specialized R packages. Batch effects in stimulated samples were corrected using CytoNorm, followed by two-step unsupervised clustering via FlowSOM and FastPG to identify immune cell clusters.

IgG Fc-glycopeptides (GLY) were profiled by affinity purification on Protein G beads, trypsin digestion, and LC-MS analysis. LacyTools was used for glycopeptide quantification, yielding subclass-specific Fc glycosylation profiles for IgG1 (24 glycopeptides) and IgG2 (14 glycopeptides); IgG3/IgG4 peptides were excluded due to allotype overlap. From these data, glycosylation traits—including fucosylation, galactosylation, sialylation, bisection, and the sialic acid/galactose ratio—were derived using standard formulas based on relative glycopeptide abundances.

Plasma cytokines (CYT) were quantified using the R&D Systems Luminex Discovery Assay Human Premixed Multi-Analyte Kit (CCL3, CCL8, CCL24, complement component C5a, CXCL10, HGF, IL-3, IL-8, IL-18, vascular endothelial growth factor, CCL2, CCL7, CCL20, CXCL9, CXCL13, IFN-γ, IL-6, IL-10, and S100A8) with reduced reagent volumes. Analytes undetectable in >40% of samples were excluded, and values below the lower limit of detection (LLOD) were imputed as half the LLOD.

Finally, these immune-profiling datasets were integrated using MOFA2 R package–an unsupervised method that learns latent factors capturing variations across datasets. Latent factors were further reduced into two dimensions using multidimensional scaling and then used as input for trajectory analysis using principal curve (princurve R package), which have previously revealed a trajectory of immune variation across the rural-urban gradient.

### Method details

#### Phage immunoprecipitation sequencing (PhIP-Seq)

PhIP-Seq assays were conducted following the protocol described by Vogl and colleagues,^19^ using the same core parameters with a few minor modifications: a 4,000-fold coverage of phages per variant and 2 μg of antibodies per reaction. The microbiota library, consisting of 244,000 variants, was mixed with two additional libraries: a 100,000-variant pool containing peptides derived from allergen and environmental compound databases,^34^ and a 13,192-variant pool primarily composed of Coronavirus antigens.^22^ Bead washing steps were automated using a Tecan Fluent 1080 liquid-handling robot. PCR amplifications were performed using primers as described previously (2021),^19^ except for PCR2, where 10-nucleotide unique dual index primers (purchased from IDT) were used.

##### PhIP-Seq data processing

Samples were sequenced with Illumina Novaseq X. Raw reads bam files were then converted to fastq format using bamtofastq (bedtools v2.31.0) and subsequently analyzed through a previously established workflow.^19^ Reads were mapped to the three antigen libraries mentioned above [refs], and raw counts for each peptide were obtained. To assess the enrichment of antibody bound peptides, peptide counts were normalized by comparing them to the oligopeptide counts in the initial library as previously done.^19^ The phage library was sequenced before immunoprecipitation and peptide counts were modeled as a generalized Poisson distribution to form the null model. For each sample, peptide enrichment significance (*p*-values) was computed while controlling for family wise error rate (FWER) with the Bonferroni method.

##### Annotation of epitopes

The agilent library peptides were run against the Uniref90^35^ database to annotate the protein and organism of origin. Some of the results from the Uniref90 database did not have a specific tax, showing up as “Bacteria” or “root”. The amino acid sequences from the non-specific agilent and the entire twist peptide libraries were analyzed with blastP against non-redundant protein sequences and the result XML files retrieved. For each epitope, the scientific names of the top 15 results (in terms of E-value) were extracted. The organism origin of the epitope was assigned to be the most common scientific name in the top 15 list. Ties were broken randomly. Manual standardization of the organism names was also carried out, for example removing strain information from the scientific name and ensuring continuity between the names derived from the Uniref90 database and blast. For example, cytomegalovirus is named “Human betaherpesvirus 5” in blast results and “Human cytomegalovirus” in the Uniref90 results. This was standardised to “Human cytomegalovirus”.

### Quantification and statistical analysis

All statistical analyses were performed in R (v4.4.1). All data points represent one biological replicate; technical replicates were not performed. No samples were omitted from analysis. All tests were two-sided.

The PhIP-Seq dataset was treated as a binary matrix indicating presence or absence of antibody against each epitope. Individuals showing significant enrichment for a particular epitope were classified as having a detected antibody response against that epitope.

Multidimensional scaling (MDS) was performed on a filtered subset of the binary PhIP-Seq dataset using Jaccard distance, where epitopes were retained if detected in at least four individuals from any one of the three residence groups. Significant pairwise differences between residence and sex groups in MDS space were assessed using PERMANOVA, as implemented in the adonis2 function of the vegan R package.^36^ Rarefied diversity and Simpson’s Index metrics were also calculated using the vegan package.

To assess whether anti-epitope detection frequencies showed a linear trend across residence groups, Cochran-Armitage trend tests were performed across all individuals and epitopes, with *p*-values adjusted using the Benjamini-Hochberg procedure to account for multiple testing. For testing linear trends across continuous data (e.g., immune parameters), orthogonal polynomial contrasts were utilized; features showing a significant linear but not quadratic trend were retained as significant.

To explore the relation between antibody repertoire features to immune profiling features, we performed two-way orthogonal partial least squares (O2PLS) integration of the PhIP-Seq and immune-profiling datasets using the OmicsPLS R package. O2PLS decomposes two data matrices into joint (co-varying), data-specific orthogonal, and residual components, modeling the shared variation between blocks. The number of joint and data-specific orthogonal components was selected by 10-fold cross-validation, minimising the mean squared prediction error. Joint-component loadings were used to identify the PhIP-Seq and immune features contributing most strongly to the shared variation between the two data types.

Statistical symbolic significance: ns P/FDR≥0.05; ∗P/FDR<0.05; ∗∗P/FDR<0.01; and ∗∗∗P/FDR<0.001.

## Supplemental information

Document S1. Figures S1–S9

Data S1. Epitopes in which antibodies were detected in at least one individual Information included is whether the organism name was derived from blastP or not, the number of individuals in which antibody was detected, the amino acid sequence and the epitope ID.

Data S2. Epitopes that were found to have a significant linear trend across the rural-urban axis Statistics from the Cochran-Armitage test results can be found, as well as the epitope ID and the protein and organism from which the epitope is from.
