# Supplementary material for: Phage-display immunoprecipitation sequencing reveals distinct antibody repertoire patterns across rural and urban populations
Source: iScience. 2026 Aug 18;29(9):117252. doi: 10.1016/j.isci.2026.117252 (PMC13508669; doi:10.1016/j.isci.2026.117252)
Supplement: Supplementary file 1 [file mmc1.pdf]

iScience, Volume *29*

Supplemental information

Phage-display immunoprecipitation sequencing reveals distinct antibody repertoire patterns across rural and urban populations

Ross F. Laidlaw, Gabriel Innocenti, Melanie Prinzensteiner, Thomas Vogl, Moustapha Mbow, Maria Yazdanbakhsh, and Mikhael D. Manurung

Supplementary information

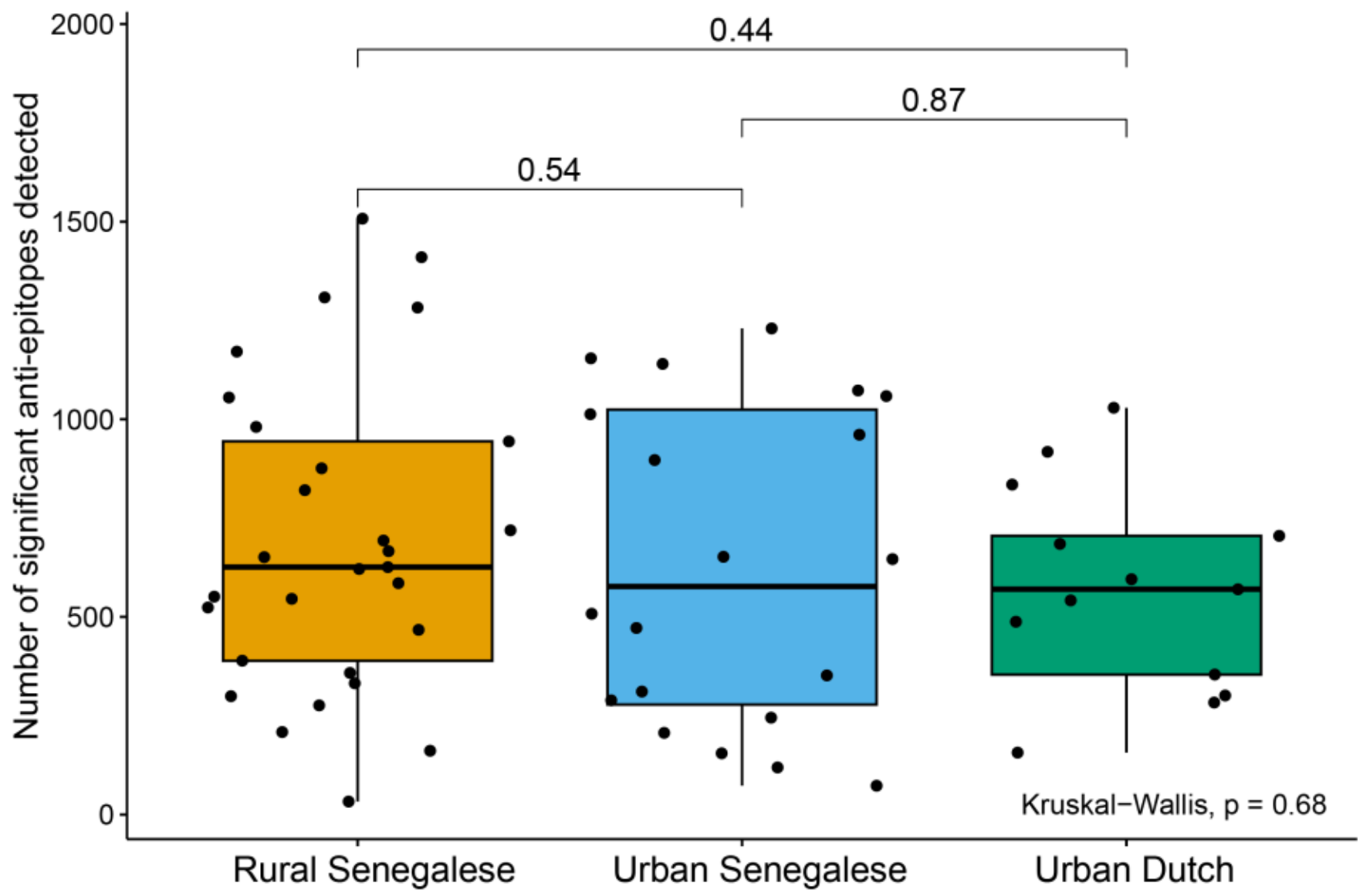

Supplementary Figure 1. Comparison of total anti-epitopes between residence areas. Total anti-epitope numbers did not differ significantly between groups. Boxplots showing the number of significant anti-epitopes identified per individual by residence group. Kruskal-Wallis and Wilcoxon Rank-Sum test *P***-**values shown.

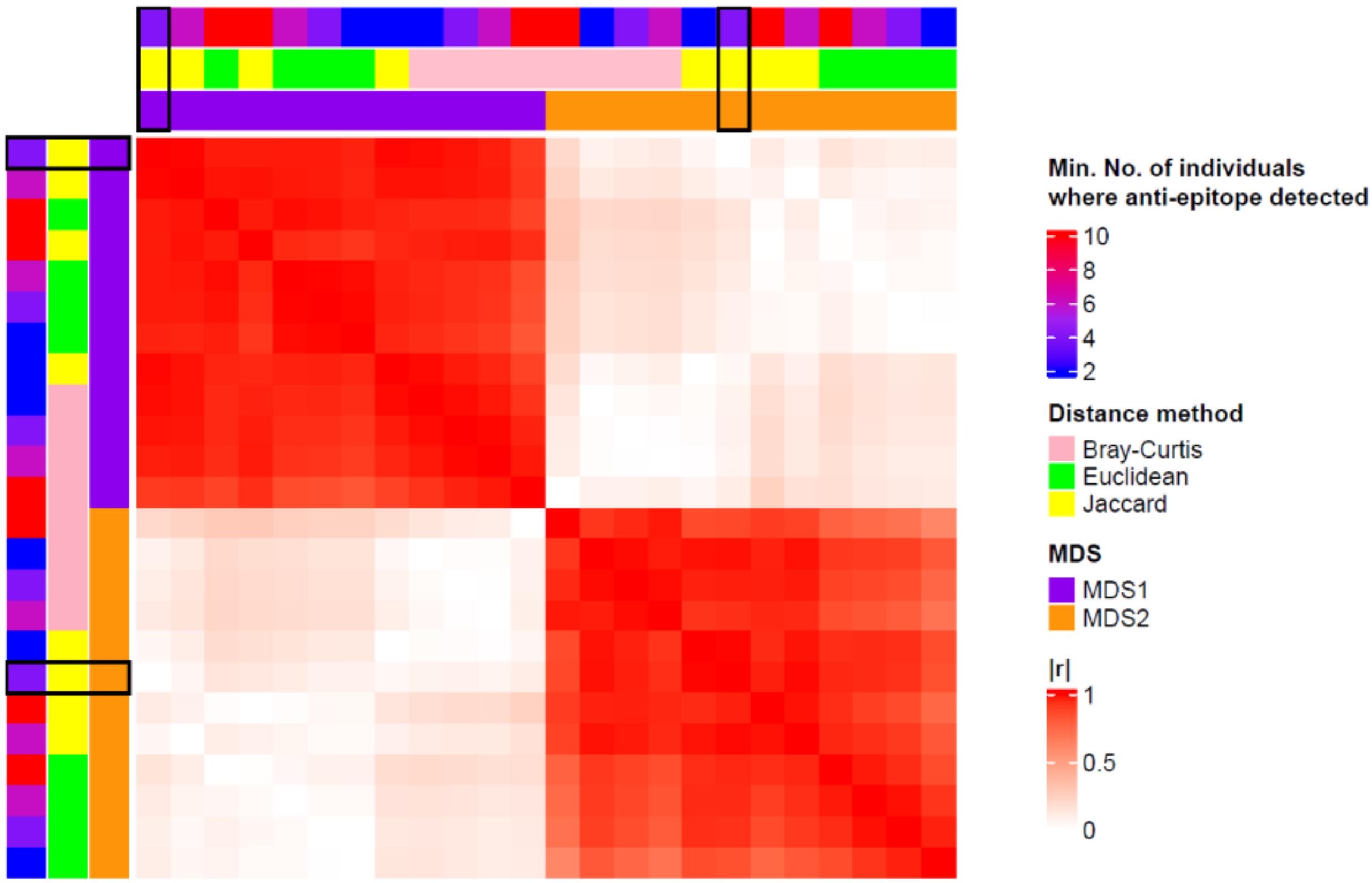

Supplementary Figure 2. Correlation of antibody repertoires across different MDS parameters. Heatmap showing the absolute Pearson r of the MDS 1 and 2 axes with different setups as to different thresholds for how many individuals an anti-epitope needs to be detected in to remain in the dataset (2, 4, 6 or 10 individuals) and the type of distance method (Bray-Curtis, Euclidean, or Jaccard) used to calculate the embeddings. The black box highlights the axes shown in Fig. 1D.

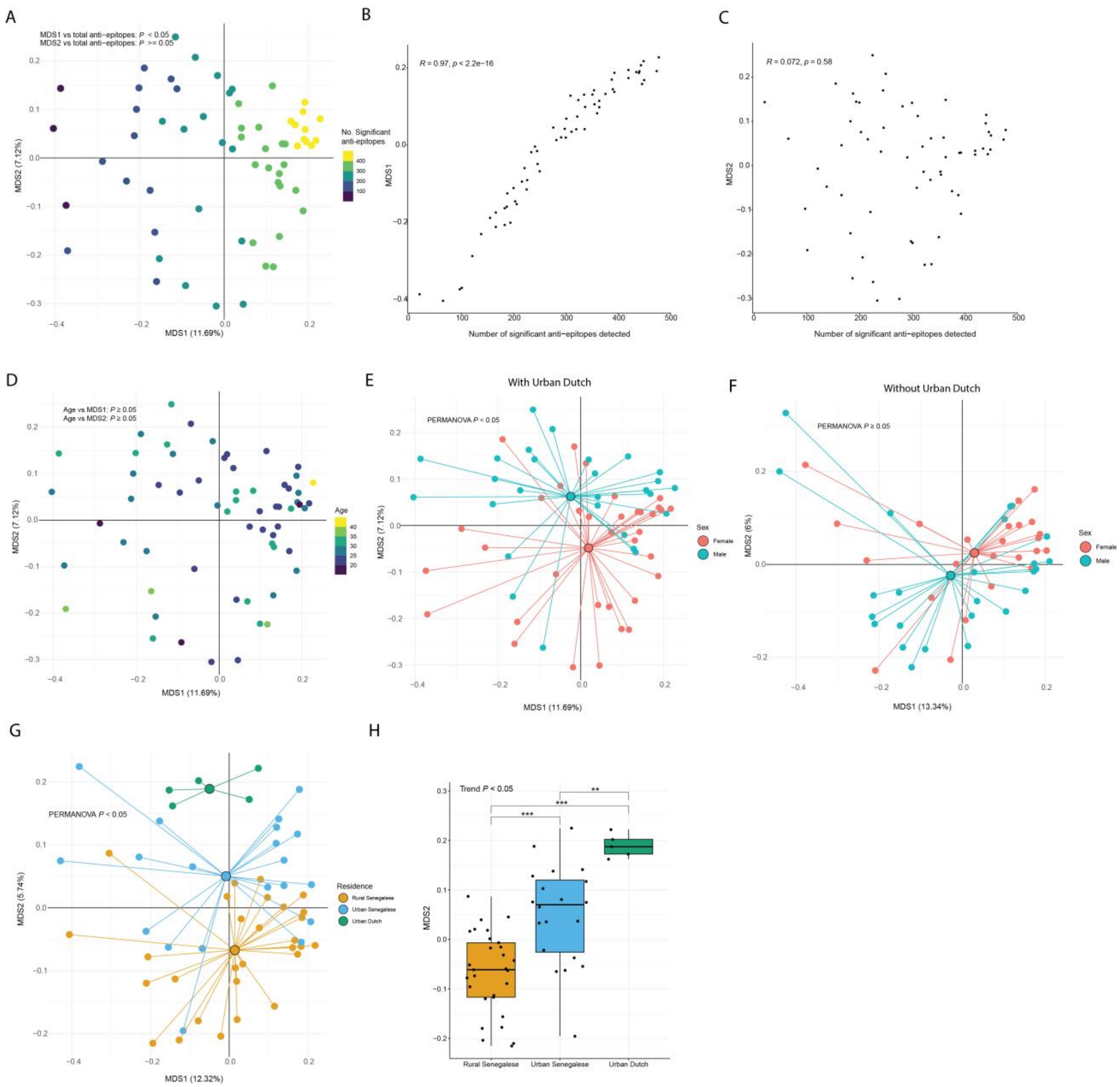

Supplementary Figure 3. Associations of MDS antibody repertoire with meta data features. MDS plot of the filtered PhIP-Seq dataset, coloured by total number of significant anti-epitopes detected (**A**), with the correlations of the MDS1 (**B**) and MDS2 (**C**) axis with total number of significant anti-epitopes detected. MDS plots coloured by age (**D**) and sex when the urban Dutch samples were included (**E**) or excluded (**F**). Panels **G** and **H** show the PCoA results when the urban Dutch group were down sampled randomly to three females (with all two males remaining). For the MDS plots, either Pearson correlation tests for MDS axes and the colouring variable or *P*-values from PERMANOVA tests to compare group centroids are shown. Boxplot in **G** shows median, quartiles, and whiskers extending 1.5× the interquartile range. Linear trend and pairwise test *P*-values shown (FDR-corrected). ^{NS}*P*/FDR≥0.05, **P*/FDR<0.05, ***P*/FDR<0.01, and ****P*/FDR<0.001

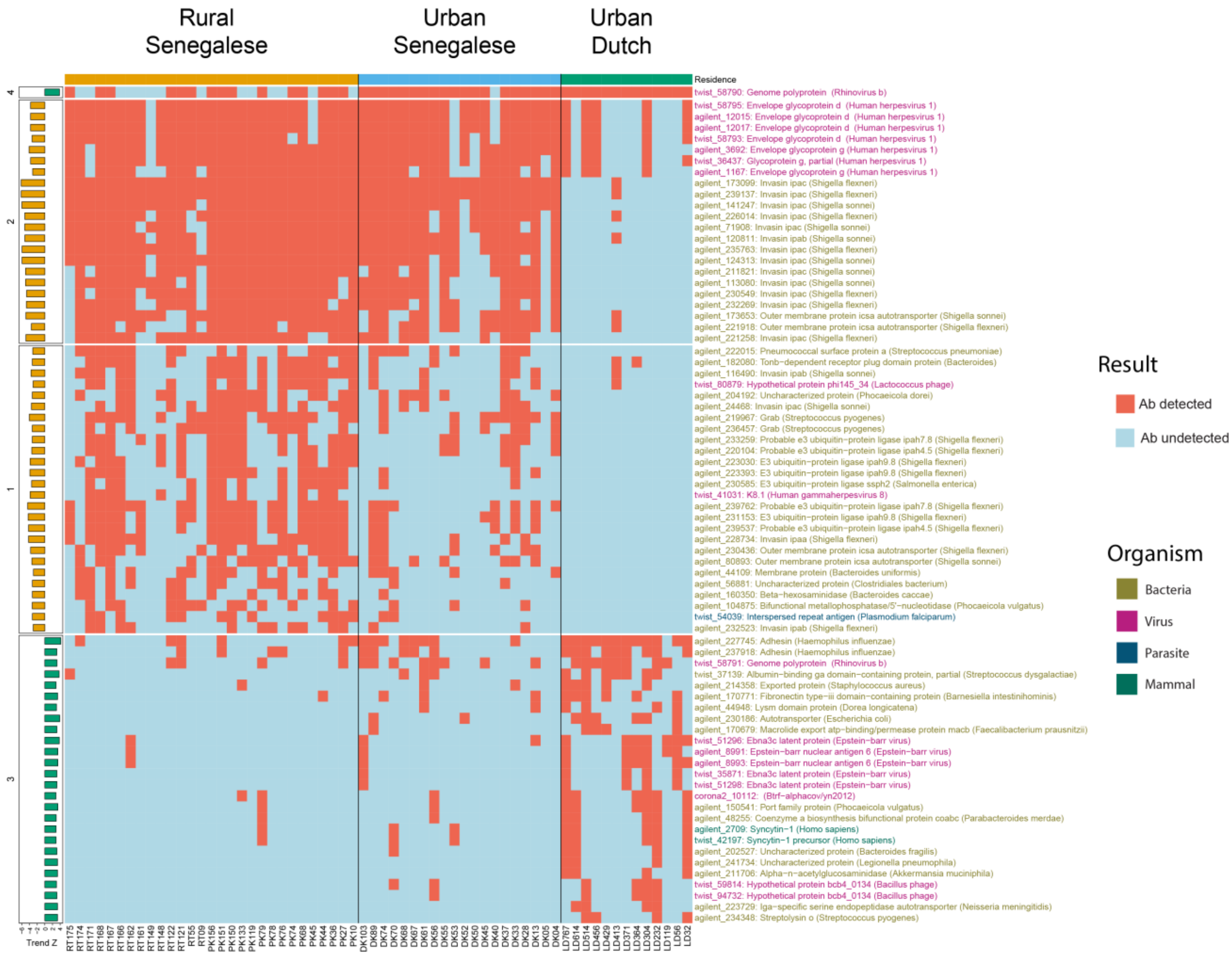

Supplementary Figure 4, related to Figure 2A. Heatmap of anti-epitope detection. Clustered heatmap showing whether antibody against a particular epitope was detected (red) or undetected (blue) in an individual. The heatmap is sliced column-wise by the residence of the individual, and row-wise by k-means clustering on the epitopes. Epitope IDs, protein and organism of origin are annotated and color coded by taxa as in Figure 2A-B. The row barplots show the trend Z value for each epitope, with each bar coloured by trend direction across the rural-urban axis.

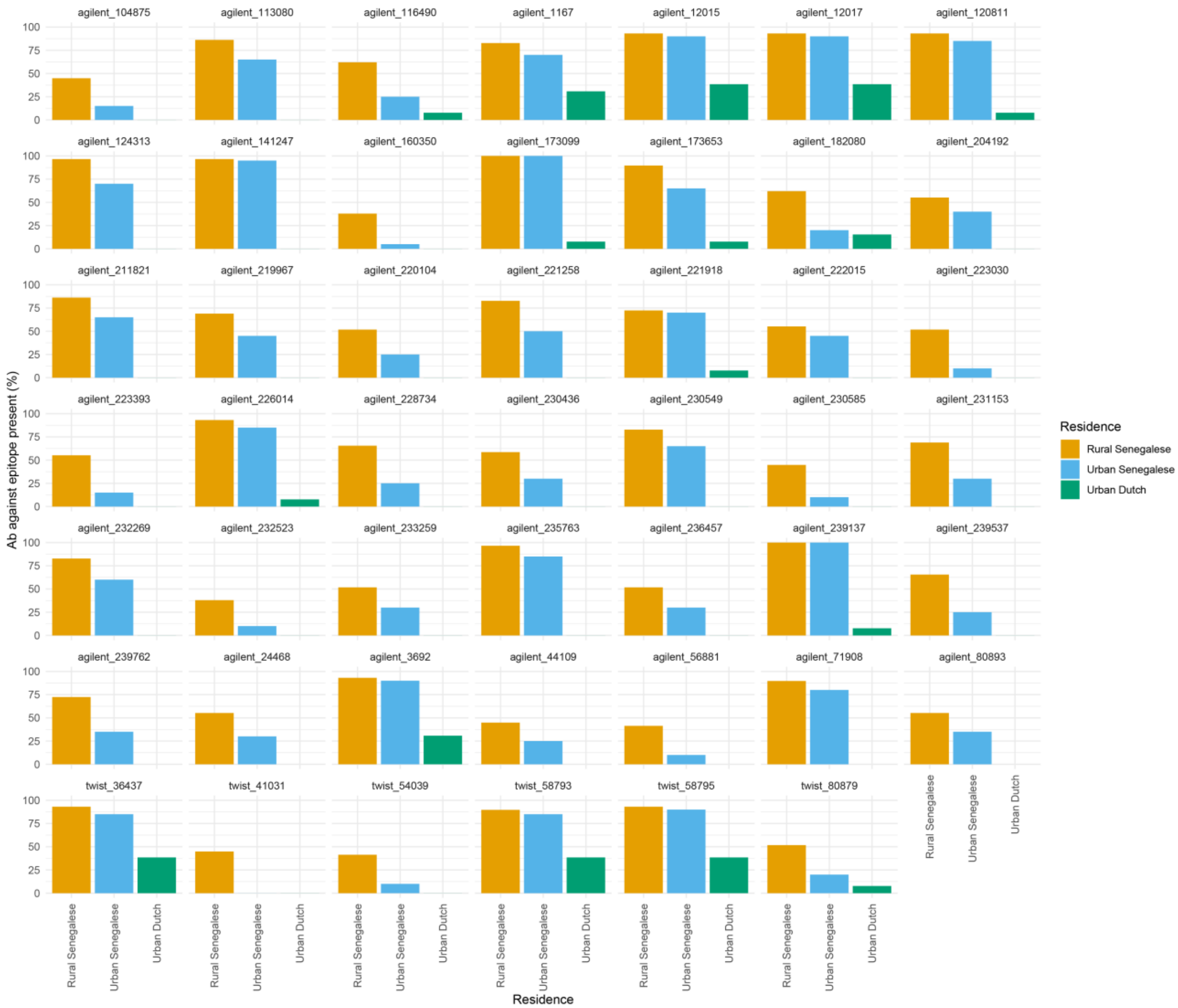

Supplementary Figure 5. Anti-epitopes associated with the Rural Senegalese end of the rural-urban axis. Barplots showing the percentage of individuals per residence group with anti-epitopes showing significant linear trends toward rural Senegalese populations across the rural-urban axis.

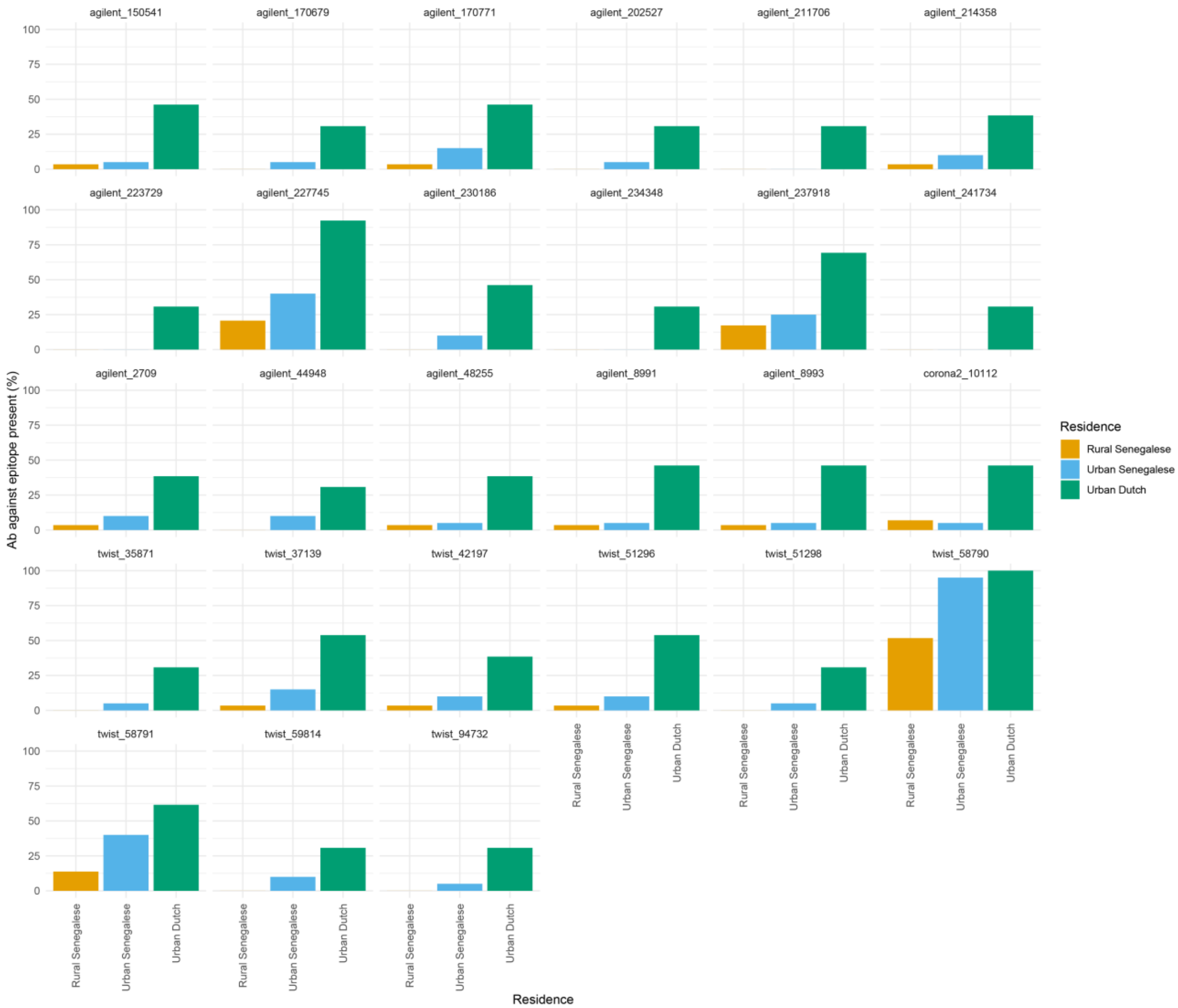

Supplementary Figure 6. Anti-epitopes associated with the Urban Dutch end of the rural-urban axis. Barplots showing the percentage of individuals per residence group with anti-epitopes showing significant linear trends toward urban Dutch populations across the rural-urban axis.

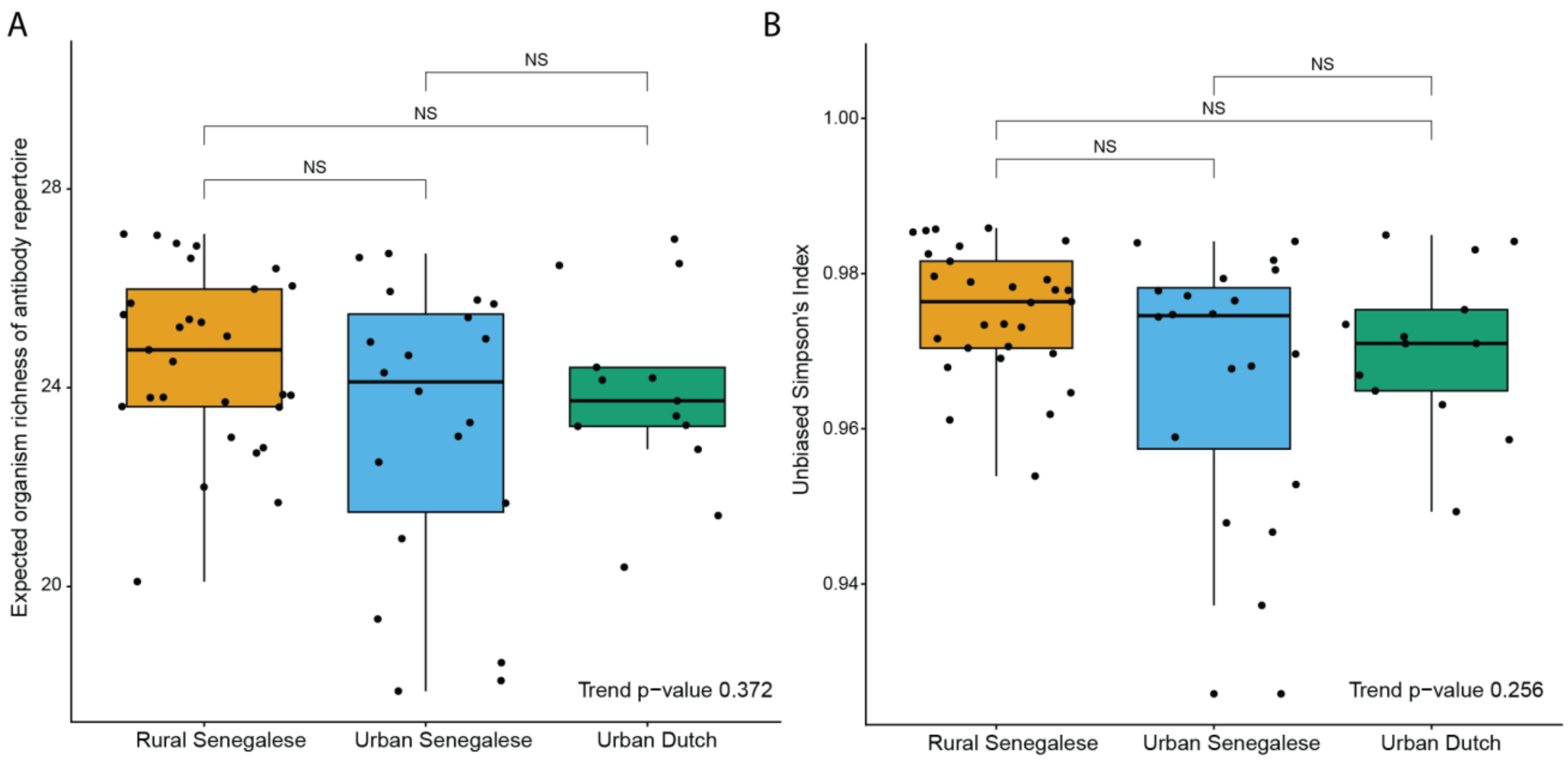

Supplementary Figure 7. Organism-level diversity of antibody repertoires does not differ significantly across residence groups, related to Figure 2. (A) Expected organism richness of the antibody repertoire, calculated using rarefaction, across rural Senegalese (n=29), urban Senegalese (n=20), and urban Dutch (n=13) participants. No significant pairwise differences were observed (Tukey post hoc test), and no significant linear trend was detected across the rural-urban axis (trend P = 0.372). (B) Unbiased Simpson's Index of the antibody repertoire at the organism level across the three residence groups. No significant pairwise differences or linear trend were observed (trend P = 0.256). Boxplots show the median (center line), interquartile range (box limits), and 1.5× interquartile range (whiskers). Individual data points are overlaid. NS, not significant.

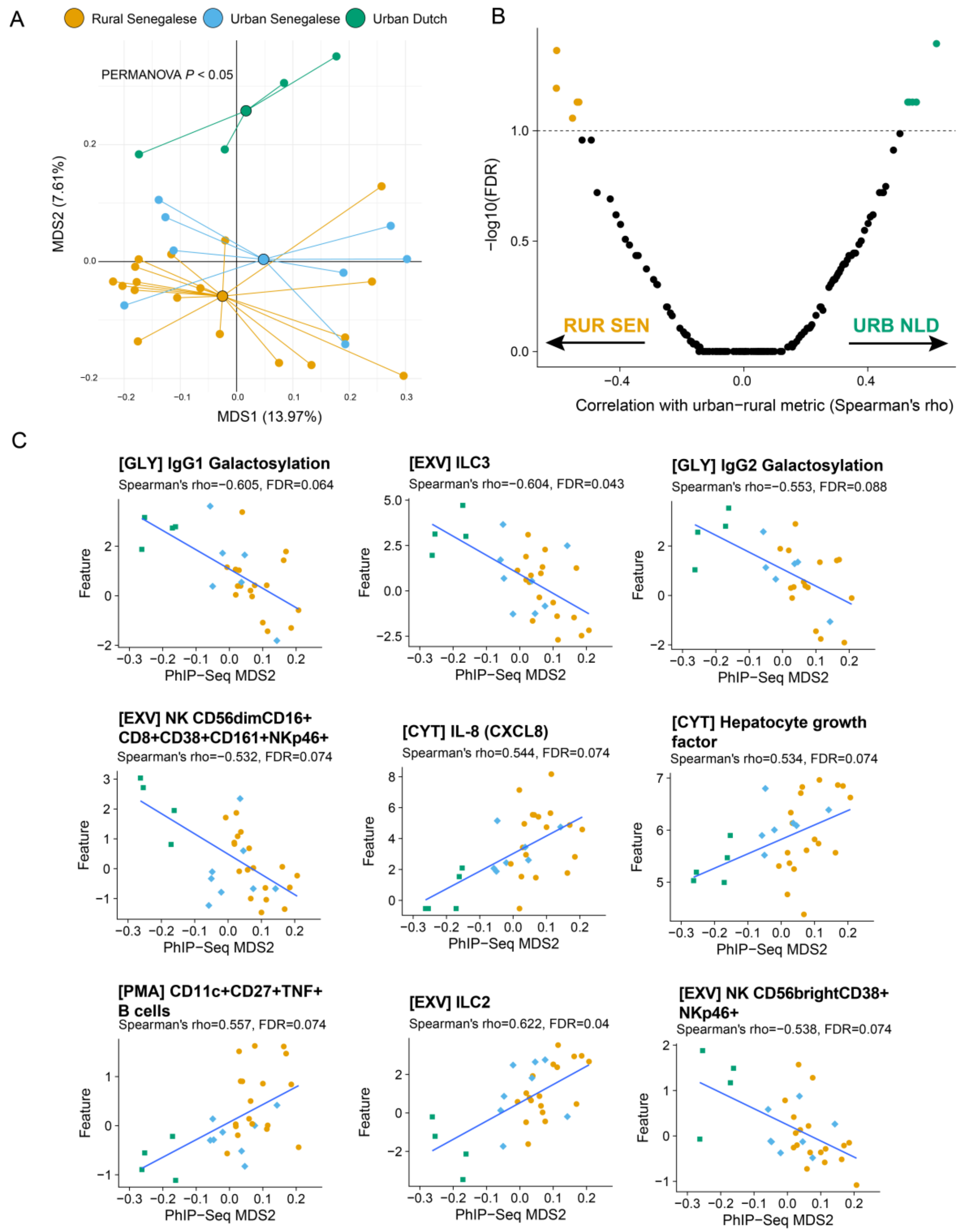

Supplementary Figure 8. Association of antibody-repertoire gradient and immune features. A, MDS plot of PhIP-Seq dataset, subsetted for individuals with corresponding high-dimensional immune-profiling datasets. **B**, Volcano plot of correlations between PhIP-Seq MDS2 trajectory with immune parameters. **C**, Scatter plot showing immune parameters with statistically significant correlation (FDR<0.1) with PhIP-Seq MDS2 trajectory.

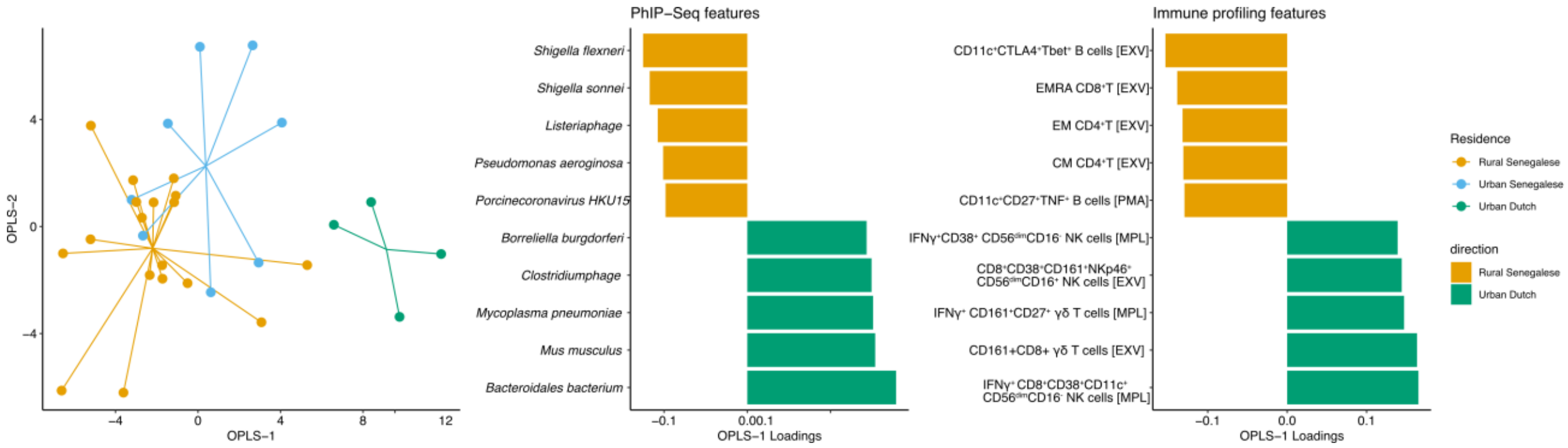

Supplementary Figure 9. O2PLS integration links antibody repertoire features to systemic immune phenotypes. Two-way orthogonal partial least squares (O2PLS) was used to model joint variation between PhIP-Seq antibody repertoire features and immune-profiling features in the 30 individuals with paired data. **Left,** sample scores on the first two joint components, coloured by residence group; lines connect each individual to their group centroid. Individuals separate along joint component 1 (OPLS-1) in accordance with residence along the rural-urban gradient. Model dimensionality (two joint, two X-orthogonal, two Y-orthogonal components) was selected by 10-fold cross-validation minimising mean squared prediction error (MSE = 1.97). **Centre and right,** features with the largest loadings on joint component 1 for the PhIP-Seq block (centre) and the immune-profiling block (right). Bars are coloured by the direction of association (rural Senegalese, orange; urban Dutch, green). Immune-feature labels indicate the source dataset in brackets: EXV, ex vivo mass cytometry; PMA, PMA/ionomycin stimulation; MPL, monophosphoryl lipid-A stimulation.
